# Placental Alkaline Phosphatase Promotes Zika Virus Replication by Stabilizing Viral Proteins through BIP

**DOI:** 10.1128/mBio.01716-20

**Published:** 2020-09-15

**Authors:** Jian Chen, Zhilu Chen, Mingbin Liu, Tianyi Qiu, Daobin Feng, Chen Zhao, Shuye Zhang, Xiaoyan Zhang, Jianqing Xu

**Affiliations:** a Shanghai Public Health Clinical Center & Shanghai Institutes of Biomedical Sciences, Shanghai Medical College, Fudan University, Shanghai, China; Washington University School of Medicine

**Keywords:** BIP, placental alkaline phosphatase, Zika virus, placental trophoblast cells

## Abstract

Zika virus (ZIKV) infection during pregnancy causes intrauterine growth defects and microcephaly, but knowledge of the mechanism through which ZIKV infects and replicates in the placenta remains elusive. Here, we found that ALPP, an alkaline phosphatase expressed primarily in placental tissue, promoted ZIKV infection in both human placental trophoblasts and astrocytoma cells. ALPP bound to ZIKV structural and nonstructural proteins and thereby prevented their proteasome-mediated degradation and enhanced viral RNA replication and virion biogenesis. In addition, the function of ALPP in ZIKV infection depends on its phosphatase activity. Furthermore, we demonstrated that ALPP was stabilized through interactions with BIP, which is the endoplasmic reticulum (ER)-resident heat shock protein 70 chaperone. The chaperone activity of BIP promoted ZIKV infection and mediated the interaction between ALPP and ZIKV proteins. Collectively, our findings reveal a previously unrecognized mechanism through which ALPP facilitates ZIKV replication by coordinating with the BIP protein.

## Abstract (precis)

ZIKV is a recently emerged mosquito-borne flavivirus that can cause devastating congenital Zika syndrome in pregnant women and Guillain-Barré syndrome in adults, but how ZIKV specifically targets the placenta is not well understood. Here, we identified an alkaline phosphatase (ALPP) that is expressed primarily in placental tissue and promotes ZIKV infection by colocalizing with ZIKV proteins and preventing their proteasome-mediated degradation. The phosphatase activity of ALPP could be required for optimal ZIKV infection, and ALPP is stabilized by BIP via its chaperone activity. This report provides novel insights into host factors required for ZIKV infection, which potentially has implications for ZIKV infection of the placenta.

## INTRODUCTION

The Zika virus (ZIKV) outbreak in South America is associated with neonatal microcephaly and atypical Guillain-Barré syndrome (1, 2). *In vivo* (3–8) and *in vitro* (9–11) studies have demonstrated that ZIKV replicates in the placenta, in the brain tissues of fetuses with microcephaly, or in placenta-derived primary cells. However, knowledge of the mechanism through which ZIKV infects and replicates in the placenta remains elusive (12, 13). ZIKV, similarly to other flaviviruses, has a single-stranded positive-sense RNA genome of approximately 11 kb that is translated into a single large polyprotein, and this polyprotein is cleaved into individual proteins at the rough endoplasmic reticulum (ER) (14–16). The ZIKV proteins form large complexes and engage in multiple and complicated functions (17). Because only 10 proteins are encoded by its small RNA genome, ZIKV is highly dependent on host factors for both its replication and the generation of virus-induced compartments involved in viral RNA (vRNA) synthesis and particle assembly (18). ZIKV replication also depends on the host proteostasis machinery for the production of functional viral proteins (19). Chaperones, including heat shock protein 70 (Hsp70) networks, are required at distinct steps of the flavivirus life cycle. Hsp70 cooperates with defined sets of cochaperones to function at various steps in the dengue virus (DENV) and Zika virus viral life cycles, including viral entry, RNA replication, and capsid assembly (20–22).

In this study, we found that a human placental alkaline phosphatase (ALPP) directly interacts with ZIKV proteins and prevents their proteasomal degradation. ALPP, which was identified in human placental tissue in the 1960s (23), is primarily expressed in placental and endometrial tissue and exhibits particularly high expression during pregnancy (24, 25), which suggests that ALPP might play an important role in the activities of ZIKV in the placenta. We further demonstrated that the phosphatase activity of ALPP may be required for ZIKV infection. Investigation of the underlying mechanism showed that ALPP is stabilized through interactions with BIP, an Hsp70 chaperone in the ER that assists in protein folding and in surveillance of misfolded proteins (26, 27).

## RESULTS

### ALPP promotes ZIKV infection in human placental trophoblast cells and astrocytes.

**FIG 1:**
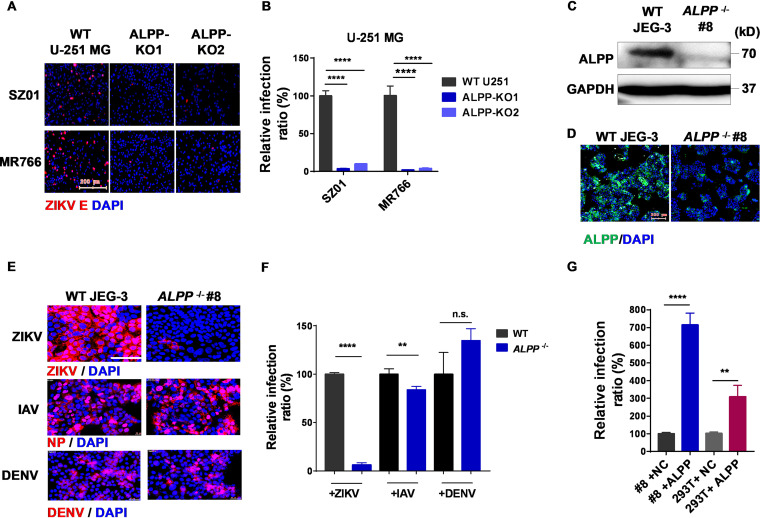
ALPP depletion prevents productive ZIKV infection in human placental trophoblast cells and astrocytes. (A and B) ZIKV infection in WT and ALPP-KO U-251 MG astrocytoma cell lines. (A) Representative immunofluorescence images from three independent experiments. MOI = 0.5. Scale bars, 200 μm. (B) Statistical analyses. Each biological replicate (*n* = 3) contained 3,000 analyzed cells. ****, *P* < 0.0001 (significantly different compared with the WT cells; two-way ANOVA with Sidak’s multiple-comparison test). (C and D) Immunoblot (C) and immunofluorescence (D) analyses of ALPP protein levels. The images shown are representative of results from three independent experiments. Scale bars, 200 μm. ALPP, green; DAPI, blue. (E and F) ZIKV, IAV, and DENV infection in WT and *ALPP*^−/−^ human placental trophoblasts (JEG-3). (E) Representative immunofluorescence images from three independent experiments. Scale bars, 100 μm. (F) Statistical analyses. Each biological replicate (*n* = 3) contained 3,000 analyzed cells. n.s., no significance; **, *P* = 0.0015; ****, *P* < 0.0001 (significantly different compared with the WT cells; two-tailed Student's *t* test). (G) ZIKV infection in ALPP-overexpressing *ALPP*^−/−^ JEG-3 cells and 293T cells. MOI = 2. Each biological replicate (*n* = 3) contained 3,000 analyzed cells. ****, *P* < 0.0001 (significantly different compared with the NC-treated cells; two-tailed Student's *t* test); **, *P* = 0.0052 (significantly different compared with the NC-treated 293T cells; two-tailed Student's *t* test). The quantitative data in this figure are shown as means ± SEMs. DAPI, blue; ZIKV E, red; NP, red; DENV, red; NC, negative control.

Using a genome-scale clustered regularly interspaced short palindromic repeats (CRISPR) single-guide RNA (sgRNA) screening approach, we previously identified host factors involved in the replication of ZIKV and the latency of HIV-1 (28, 29). In this study, we used the same approach to achieve gene knockout (KO) in a human astrocytoma cell line (U-251 MG cells), and cells resistant to ZIKV infection were selected for sequencing (see Fig. S1A in the supplemental material). We designed CRISPR/Cas9 sgRNAs that target each of the enriched hits from our primary screen and confirmed that ALPP is a key host factor for ZIKV infection in U-251 MG cells. ALPP, a human placenta-enriched alkaline phosphatase, is highly expressed during pregnancy (Fig. S1B and C) (24, 25). This indicates that ALPP may play a role in ZIKV infection in the placenta. Therefore, we disrupted the *ALPP* gene in a placental trophoblast cell line (JEG-3) and the U-251 MG cell line via CRISPR/Cas9 editing using two sgRNAs that separately target the second and fourth exons of the *ALPP* gene. JEG-3 and U-251 MG cells were selected as ZIKV target cells to recapitulate the intrauterine infection events that occur in the placenta and the pathogenesis of ZIKV in the fetal brain, respectively (21, 28). Interestingly, we observed a substantial reduction in ZIKV infection in ALPP-KO U-251 cells (Fig. 1A and B) and ALPP-KO JEG-3 cells (Fig. S2A and B). In addition, ALPP knockout in HK2 cells, an immortalized proximal tubule epithelial cell line, also reduced ZIKV infection (Fig. S2C and D). We then generated an ALPP-KO JEG-3 single-cell clone (*ALPP*^−/−^, clone 8) and validated the efficiency of the KO of ALPP by the sgRNA through immunoblot and immunofluorescence analyses (Fig. 1C and D). We found that the derived cell line (*ALPP*^−/−^, clone 8) was resistant to ZIKV infection but not to influenza A virus (IAV) or dengue virus (DENV) infection (Fig. 1E and F). Depletion of ALPP in JEG-3 cells significantly attenuated ZIKV infection at different infection doses (Fig. S3A and B), significantly enhanced cell viability after ZIKV infection, and reduced ZIKV-induced cytopathic effects (CPEs) (Fig. S3C to F). Furthermore, ectopic expression of ALPP in *ALPP*^−/−^ JEG-3 cells rescued the susceptibility of these cells to ZIKV infection (Fig. 1G). Similarly, overexpression of ALPP in HEK 293T cells, a cell line with minimal expression of ALPP, significantly enhanced the susceptibility of these cells to ZIKV infection (Fig. 1G). Together, these results demonstrate that ALPP may play an essential role in ZIKV infection.

10.1128/mBio.01716-20.1 FIG S1 Expression level of ALPP in different human tissues. (The data were obtained from the updated Expression Atlas, which is an integrated database of gene and protein expression in humans, animals and plants [I. Papatheodorou, P. Moreno, J. Manning, A. Muñoz-Pomer Fuentes, et al., Nucleic Acids Res 48(D1):D77–D83, 2020, https://doi.org/10.1093/nar/gkz947]). (A) Hits of CRISPR-Cas9 screening in U-251 MG cells after ZIKV infection. (B) ALPP mRNA expression level in different organs. TPM, transcripts per million (kilobases). (C) Examples of immunohistochemical (IHC) staining of ALPP in human tissues. (Left to right) HPA038764, female, age 30, placenta (T-88100); HPA038764, female, age 41, kidney (T-71000); HPA051699, female, age 84, colon (T-67000); HPA038764, female, age 54, liver (T-56000); HPA038764, female, age 64, cerebral cortex (T-X2020); HPA038765, female, age 53, lymph node (T-08000). ALPP, brown. Download FIG S1, TIF file, 1.2 MB. Copyright © 2020 Chen et al. 2020 Chen et al. This content is distributed under the terms of the Creative Commons Attribution 4.0 International license.

10.1128/mBio.01716-20.2 FIG S2 ALPP depletion prevents ZIKV infection in JEG-3 and HK2 cells. (A and B) ZIKV infection in WT and *ALPP*^−/−^ JEG-3 cells. (A) Representative immunofluorescence images from three independent experiments. Scale bars, 100 μm. (B) Statistical analyses. Each biological replicate (*n* = 4) contained 3,000 analyzed cells. ****, *P* < 0.0001; *, *P* = 0.0197 (significantly different compared with the WT cells; two-way ANOVA with Sidak’s multiple-comparison test). c and d, ALPP knockout prevents ZIKV infection at various viral doses. (C and D) ZIKV infection in WT and ALPP-KO HK2 cells. (C) Representative immunofluorescence images from three independent experiments. Scale bars, 50 μm. (D) Statistical analyses. Each biological replicate (*n* = 3) contained 3,000 analyzed cells. ****, *P* < 0.0001 (significantly different compared with the WT cells; two-way ANOVA with Sidak’s multiple-comparison test). Download FIG S2, TIF file, 2.6 MB. Copyright © 2020 Chen et al. 2020 Chen et al. This content is distributed under the terms of the Creative Commons Attribution 4.0 International license.

10.1128/mBio.01716-20.3 FIG S3 ALPP depletion prevents ZIKV infection and reduces ZIKV-induced cell death after infection with various viral doses. (A and B) ALPP depletion prevents ZIKV infection. (A) Representative immunofluorescence images from three independent experiments. Scale bars, 100 μm. (B) Statistical analyses. Each biological replicate (*n* = 4) contained 3,000 analyzed cells. ****, *P* < 0.0001 (significantly different compared with the WT cells; two-way ANOVA with Sidak’s multiple-comparison test). (C) ALPP depletion exerted a minimal effect on the proliferation of JEG-3 cells over a period of 75 h. (D) ALPP depletion prevented ZIKV-induced cytopathic effects (CPEs) 48 h post-MR766 infection at various viral doses. Scale bars, 200 μm. (E and F) ALPP depletion prevented cell death induced by infection with ZIKV at various viral doses. (E) ZIKV MR766 infection. (F) ZIKV SZ01 infection. MOI = 2 or MOI = 10. The data are shown as means of results from two biological replicates (*n* = 2). Download FIG S3, TIF file, 1.8 MB. Copyright © 2020 Chen et al. 2020 Chen et al. This content is distributed under the terms of the Creative Commons Attribution 4.0 International license.

### ALPP is essential for Zika vRNA synthesis and virion release.

**FIG 2:**
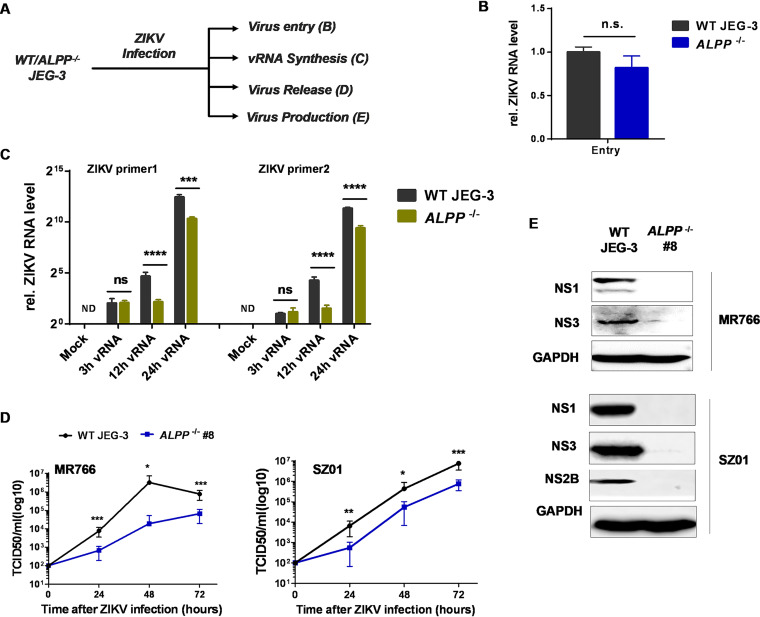
ALPP functions at multiple steps in the ZIKV infectious life cycle. (A) Schematic of ZIKV infection experiments comparing WT and *ALPP*^−/−^ JEG-3 cells. Capital letters in parentheses correspond to panels B to E of the figure. (B) Detection of ZIKV vRNA by RT-qPCR. Statistical analyses of internalized ZIKV were performed; MOI = 100. The data were obtained from four biological replicates (*n* = 4). n.s., no significance (*P* = 0.1934; two-tailed Student's *t* test). (C) Detection of intracellular ZIKV vRNA at the indicated times after ZIKV infection by RT-qPCR. MOI = 0.5. The data were obtained from four biological replicates (*n* = 4). The significant differences between WT and *ALPP*^−/−^ JEG-3 cells at each time point were determined by two-way ANOVA with Sidak’s multiple-comparison test. ****, *P* < 0.0001; n.s., no significance; ND, not detected. (D) ZIKV MR766 (left) and SZ01 (right) infectious particles produced from WT and *ALPP*^−/−^ JEG-3 cells at the indicated time after ZIKV infection. MOI = 0.5. The data were obtained from at least three biological replicates (*n* ≥ 3). Significant differences between WT and *ALPP*^−/−^ cells at each time point were determined by two-way ANOVA with Sidak’s multiple-comparison test. *, *P* < 0.05; **, *P* < 0.01; ***, *P* < 0.001. TCID_50_, 50% tissue culture infective doses. (E) Production of ZIKV MR766 (top) and SZ01 (bottom) proteins in WT and *ALPP*^−/−^ JEG-3 cells infected with ZIKV for 24 h. MOI = 0.5. The quantitative data in this figure are shown as means ± SEMs.

To investigate the underlying mechanisms, we sought to determine the step in the ZIKV life cycle at which ALPP exerts its effect (Fig. 2A). Because ALPP is a glycosylphosphatidylinositol (GPI)-anchored protein that is expressed mainly in the plasma membrane and ER, we first determined whether ALPP functions as an entry receptor for ZIKV at the plasma membrane. We performed a viral entry assay using JEG-3 and HK-2 cells as previously described (28). Although ALPP depletion did not affect the entry of ZIKV (Fig. 2B; see also Fig. S4A), ZIKV vRNA synthesis was significantly reduced in *ALPP*^−/−^ JEG-3 cells at 12 and 24 h postinfection (Fig. 2C), which suggested that ALPP likely functions at postentry steps. Furthermore, we analyzed the production of ZIKV SZ01 and MR766 virions from infected wild-type (WT) and *ALPP*^−/−^ JEG-3 cells over an infection period of 72 h and found that ALPP depletion markedly reduced the release of ZIKV virions (Fig. 2D; see also Fig. S4B). Consistent with the results from JEG-3 cells, the numbers of ZIKV plaque formation units were also significantly reduced in the supernatant of ALPP-KO U-251 MG cells (Fig. S4C). Immunoblot analyses showed that expression of ZIKV proteins, including NS1, NS2B, and NS3, was nearly absent in *ALPP*^−/−^ JEG-3 cells 24 h postinfection relative to that in WT JEG-3 cells (Fig. 2E). These results further suggest that ALPP is essential for vRNA synthesis and productive ZIKV infection in human placental trophoblasts and astrocytes.

10.1128/mBio.01716-20.4 FIG S4 ALPP knockout prevents ZIKV infection but does not affect ZIKV entry. (A) Assay of ZIKV MR766 entry in WT and ALPP-KO HK2 cells. Three biological replicates were examined in each experiment. The data are shown as means ± SEMs (*n* = 3). The significant differences between the WT and ALPP-KO results were determined by Student’s *t* test (n.s., no significance). (B) *ALPP*^−/−^ restricts ZIKV SZ01 replication in JEG-3 cells. The data were collected from at least three biologically independent experiments and are shown as means ± SEMs (*n* ≥ 3). The significant differences between the WT and *ALPP*^−/−^ results were determined by two-way ANOVA with the Sidak multiple-comparison test. ****, *P* < 0.0001. (C) *ALPP*^−/−^ restricts ZIKV MR766 replication in U-251 MG cells. Viral plaques were allowed to form on Vero cells under a liquid medium overlay. The plaques were fixed and stained with Giemsa stain at 5 days postinfection (d.p.i.). Download FIG S4, TIF file, 1.8 MB. Copyright © 2020 Chen et al. 2020 Chen et al. This content is distributed under the terms of the Creative Commons Attribution 4.0 International license.

### ALPP stabilizes viral proteins during ZIKV infection.

**FIG 3:**
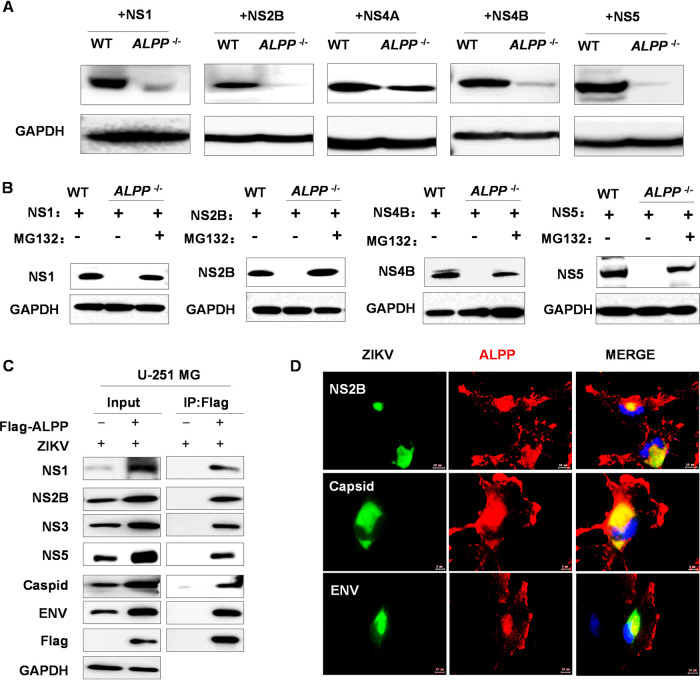
ALPP directly interacts with ZIKV proteins and prevents their proteasomal degradation. (A and B) Immunoblot analyses of the ZIKV protein levels. WT and *ALPP*^−/−^ JEG-3 cells were transiently transfected with pCMV-ZIKV nonstructural proteins for 24 h and treated without (A) or with (B) MG132 (10 μM) for an additional 12 h. (C) Immunoblot (left) and immunoprecipitation (right) analyses of lysates from WT and *ALPP*-overexpressing U-251 MG cells infected with ZIKV for 24 h. (D) Immunofluorescence analyses of the colocalization between ALPP and ZIKV proteins. Flag-ALPP U-251 MG cells were infected with ZIKV for 24 h (MOI = 2). Scale bars, 5 μm (middle row) and 10 μm (top and bottom rows). ALPP, red; ZIKV C, ENV, or NS2B, green.

Following ZIKV entry, viral (+)RNAs are released and then translated to produce viral proteins (30, 31). The nonstructural proteins form replication complexes on modified ER membranes and transcribe genomic (+)RNA into a negative-strand RNA, which serves as the template for the production of progeny (+)RNAs. Because the replication of (+)RNA viruses involves numerous interactions between components from the virus (RNA and proteins) and the host (proteins, membranes, and lipids), we hypothesized that ALPP might promote ZIKV vRNA synthesis by participating in the initial production of ZIKV proteins. We examined whether the expression levels of nonstructural proteins, including components of the viral replicase (NS2B, NS4A, NS4B, and NS5) and the assembly complex (NS1) (32), were dependent on ALPP. We transiently overexpressed the nonstructural proteins of ZIKV in WT and *ALPP*^−/−^ JEG-3 cells. Interestingly, we found significant reductions in the levels of ZIKV NS1, NS2B, NS4B, and NS5 in *ALPP*^−/−^ JEG-3 cells (Fig. 3A) but minimal reduction in the NS4A expression level, which suggests that ALPP directly affects individual ZIKV proteins even in the absence of integral components of the viral machinery. In contrast, the protein levels of IAV NP and PB2 in *ALPP*^−/−^ JEG-3 cells were not reduced compared to those in WT cells (Fig. S5A). Notably, treatment of *ALPP*^−/−^ JEG-3 cells with MG132, a proteasome inhibitor, rescued the expression of NS1, NS2B, NS4B, and NS5 (Fig. 3B). These data indicate that in the absence of ALPP, the nonstructural proteins of ZIKV are targeted for degradation, which suggests that ALPP actively stabilizes these proteins during ZIKV infection.

10.1128/mBio.01716-20.5 FIG S5 ALPP specifically interacts with ZIKV proteins. (A) Immunoblot analyses of influenza A virus (IAV) PB2 and NP levels. pCMV-PB2 and NP were transiently transfected in WT and *ALPP*^−/−^ JEG-3 cells, and the cells were harvested 24 h posttransfection. (B) Immunoblot analyses of the ALPP levels in U-251 MG cells. Cell clones stably expressing ALPP were sorted using FACS analysis. (C) Immunoblot analyses of the ALPP levels in 293T cells. (D) Immunoprecipitation with Flag-ALPP as bait followed by immunoblotting with anti-Flag, anti-ZIKV NS5, or anti-ZIKV envelope (ENV) antibodies. pCMV-ZIKV NS5 (left) and ENV (right) were transiently transfected in Flag-ALPP and Flag control cells for 24 h. Download FIG S5, TIF file, 0.3 MB. Copyright © 2020 Chen et al. 2020 Chen et al. This content is distributed under the terms of the Creative Commons Attribution 4.0 International license.

To corroborate the hypothesis that ALPP could promote ZIKV replication by stabilizing viral replicase to prevent its degradation, we subsequently examined the observations mentioned above during ZIKV infection. We established a U-251 MG cell line stably expressing Flag-tagged ALPP, isolated a cell clone (clone 15) with a high level of ALPP expression by fluorescence-activated cell sorter (FACS) analysis (Fig. S5B), and then performed coimmunoprecipitation experiments in the context of ZIKV infection. Immunoblot analyses revealed that ALPP interacted with the ZIKV nonstructural proteins NS1, NS2B, NS3, and NS5 and with the structural proteins capsid (C) and envelope (ENV) (Fig. 3C). Moreover, ALPP bound to the NS5 and envelope (E) proteins after the corresponding genes were transiently introduced into the cells (Fig. S5D). Immunofluorescence analysis of ZIKV-infected Flag-ALPP-positive cells showed that cytoplasmic ALPP colocalized with ZIKV NS2B, capsid, and ENV (Fig. 3D). Taken together, these results suggest that ALPP plays an important role in ZIKV infection through its direct interaction with ZIKV proteins.

**FIG 4:**
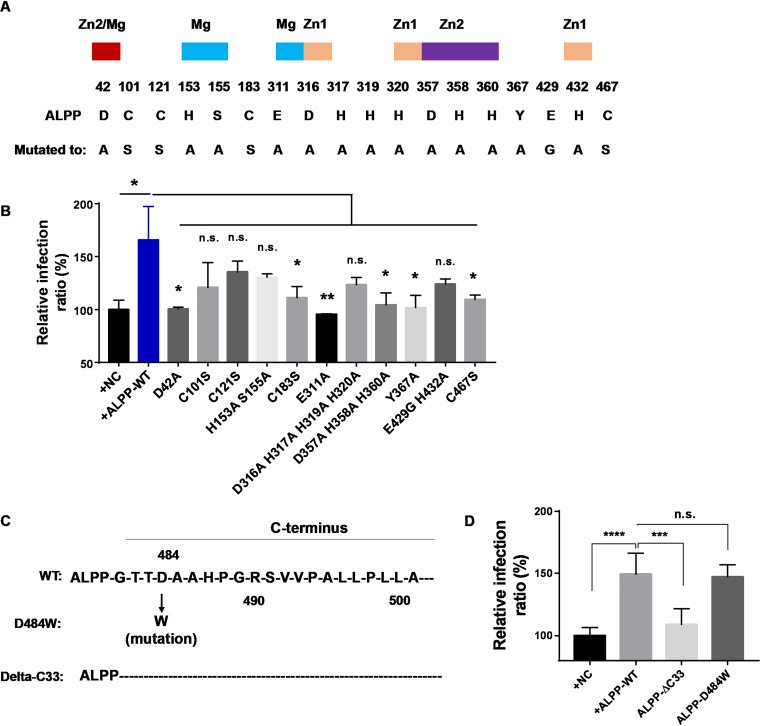
The catalytic activity and the C terminus of ALPP are needed for its function in promoting ZIKV infection. (A) Residues that have been mutagenized in ALPP activity sites. A colored box over the residue number indicates that it is a ligand to the active site Mg (blue), Zn1 (yellow), Zn2 (purple), or both Zn2 and Mg (red). (B) Statistical analyses of ZIKV infection in wild-type ALPP- or alkaline phosphatase-impaired ALPP mutant-overexpressing cells. Each biological replicate (*n* ≥ 3) contained 3,000 analyzed cells. MOI = 0.5. n.s., no significance; *, *P* < 0.05; **, *P* < 0.01 (significantly different compared with the wild-type ALPP-overexpressing cells; two-tailed Student's *t* test). (C) Structures of the wild-type, mutant, and delta-C-terminal ALPP used in this study. (D) Statistical analyses of ZIKV infection in cells overexpressing wild-type or mutant ALPP. Each biological replicate (*n* ≥ 3) contained 3,000 analyzed cells. MOI = 0.5. n.s., no significance; ***, *P* < 0.001; ****, *P* < 0.0001 (significantly different compared with the wild-type ALPP-treated cells; two-tailed Student's *t* test).

To gain more insight into the functional mechanism of ALPP, we assessed whether the catalytic activity of ALPP is required for promoting ZIKV infection. We constructed ALPP enzymatic mutants (33), including D42A, C101S, C121S, H153A, S155A, C183S, E311A, D316A, H317A, H319A, H320A, D357A, H358A, H360A, Y367A, H432A, and C467S (Fig. 4A), and found that these mutations at catalytic-activity sites of ALPP significantly affected the role of ALPP in ZIKV infection (Fig. 4B). Thus, the catalytic activity of ALPP is important for its role in promoting ZIKV infection.

### BIP associates with ALPP and is essential for ZIKV infection.

To determine whether additional host factors participate in the ALPP-ZIKV interactome, we performed a coimmunoprecipitation experiment with uninfected Flag-ALPP-overexpressing U-251 MG cells and pulled down a distinctive 78-kDa protein (Fig. S6A). Mass spectrometry analyses identified this protein as BIP (also called GRP78), an ER-resident Hsp70 chaperone that assists in protein folding and in the surveillance of misfolded proteins (27) (see Table S1B in the supplemental material). ALPP is directed to the ER via an N-terminal cleavable signal peptide. At its extreme C-terminal end, a hydrophobic stretch of amino acids enables attachment of the GPI anchor and is subsequently cleaved off. A previous study (26) demonstrated that BIP binds to ALPP specifically via its C-terminal hydrophobic stretch. BIP thus binds only the proform of ALPP and not the mature GPI-linked form. To assess whether the GPI anchor or the hydrophobic stretch at the C terminus is required for the function of ALPP in ZIKV infection, we constructed an ALPP-C-terminus-deleted protein (ALPP-ΔC33) and an ALPP-D484W mutant that rendered a loss of the GPI linker (Fig. 4C). Deletion of 33 amino acids at the C terminus significantly inhibited the function of ALPP in ZIKV infection (Fig. 4D). In contrast, the GPI-defective mutant (D484W) did not affect the function of ALPP in ZIKV infection (Fig. 4D), which indicated that the addition of a GPI anchor (mature form) is not required for ALPP function in ZIKV infection.

10.1128/mBio.01716-20.6 FIG S6 BIP knockout does not affect ZIKV entry but reduces ZIKV protein levels. (A) Immunoprecipitation with Flag-ALPP as bait followed by silver and Coomassie staining analyses. Flag-ALPP and untreated Flag control cells were used. (B) Immunoprecipitation of Flag-ALPP and Flag control JEG-3 cells with Flag-ALPP as bait followed by immunoblotting with anti-Flag or anti-BIP antibodies. (C) RNA related to ZIKV attachment and entry in WT and BIP-KO JEG-3 cells after incubation with ZIKV at the indicated MOI. RNA expression was determined by RT-qPCR for each biological replicate (*n* = 4). n.s., no significance. The significant differences between WT and *ALPP*^−/−^ cells at each time point were determined by two-way ANOVA with the Sidak multiple-comparison test. (D) Immunoblot analyses of ZIKV NS3 and ENV levels. pCMV-ZIKV NS3 (left) and ENV (right) were transiently expressed in WT or *BIP*^−/−^ U-251 MG cells, and the cells were harvested 24 h posttransfection. The data are representative of results from two independent replicates. Download FIG S6, TIF file, 0.4 MB. Copyright © 2020 Chen et al. 2020 Chen et al. This content is distributed under the terms of the Creative Commons Attribution 4.0 International license.

10.1128/mBio.01716-20.9 TABLE S1 (A) List of primers. (B) Proteins identified by liquid chromatography-mass spectrometry (LC-MS). Download Table S1, DOCX file, 0.02 MB. Copyright © 2020 Chen et al. 2020 Chen et al. This content is distributed under the terms of the Creative Commons Attribution 4.0 International license.

**FIG 5:**
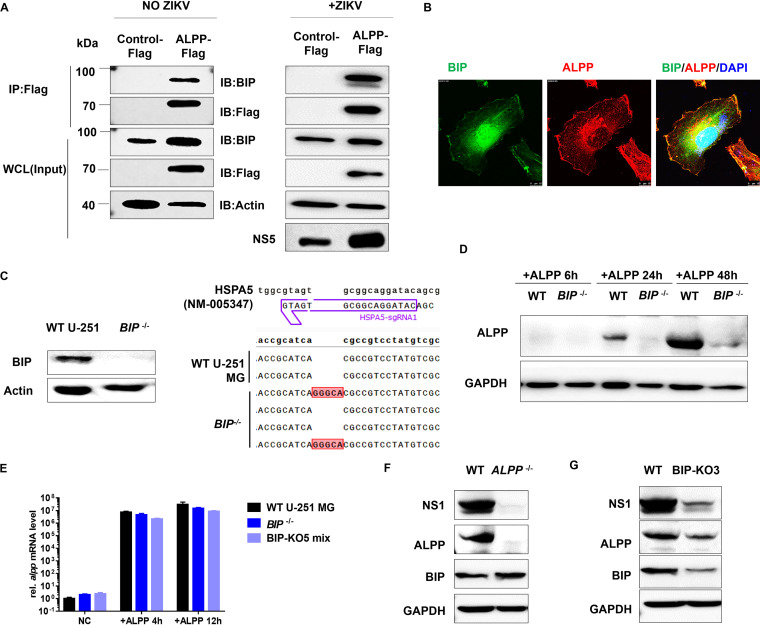
ALPP interacts with BIP directly and is stabilized by BIP. (A) Immunoprecipitation (IP) with Flag-ALPP as bait followed by immunoblotting (IB) with anti-Flag or anti-BIP antibodies. WCL, whole-cell lysate. (B) Immunofluorescence confocal microscopy of U-251 MG cells stably expressing Flag-ALPP. The cells were stained with anti-ALPP antibody (red) or anti-BIP antibody (green). Scale bars, 10 μm. (C) Immunoblot analyses of the BIP protein levels (left) and sequences of mutations in *BIP*^−/−^ U-251 MG clones (right). BIP was depleted using CRISPR/Cas9. (D) Immunoblot analyses of the ALPP protein levels in WT and *BIP*^−/−^ U-251 MG cells transiently transfected with pcDNA3.1-ALPP for 6, 24, or 48 h. (E) RT-qPCR analyses of the ALPP mRNA levels. WT and *BIP*^−/−^ U-251 MG cells were transiently transfected with pcDNA3.1-ALPP for 4 and 12 h. The data were obtained from four biological replicates (*n* = 4). (F) Immunoblot analyses of the ALPP, NS1, and BIP protein levels in WT and *ALPP*^−/−^ JEG-3 cells transiently transfected with pCMV-NS1 for 24 h. (G) Immunoblot analyses of the NS1, ALPP, and BIP protein levels. WT and BIP-KO JEG-3 cells were transiently transfected with pCMV-NS1 for 24 h. The quantitative data in this figure are shown as means ± SEMs.

To confirm the interaction between BIP and ALPP, we immunoprecipitated ectopically expressed Flag-tagged ALPP and found that BIP was indeed pulled down both in the absence (Fig. 5A, left) and in the presence (Fig. 5A, right) of ZIKV infection. This finding was further corroborated by those in Flag-ALPP-293T cells (Fig. S5C and S6A) and Flag-ALPP-JEG-3 cells (Fig. S6A and B). Furthermore, the immunofluorescence of Flag-tagged ALPP in U-251 MG cells revealed a reticular pattern of ALPP localization that overlapped that of BIP (Fig. 5B). These results indicate the participation of BIP in the ALPP-viral protein interactome.

To evaluate the role of BIP, we deleted BIP in U-251 MG cells via CRISPR/Cas9 analysis (Fig. 5C) and observed a significant reduction in ALPP expression in *BIP*^–/–^ U-251 MG cells (Fig. 5D). However, the *ALPP* mRNA levels were unaffected in *BIP*^–/–^ cells (Fig. 5E). To further dissect the influence of ALPP on BIP, we examined the expression of BIP in WT and *ALPP*^–/–^ JEG-3 cells and found that ALPP depletion had no effect on BIP expression (Fig. 5F). However, despite their normal expression of BIP, ALPP-depleted cells showed significantly reduced ZIKV NS1 protein expression (Fig. 5F). Moreover, the knockdown of BIP decreased the endogenous ALPP level and reduced ZIKV NS1 protein expression (Fig. 5G).

Cochaperones, such as DNAj-like proteins, are known to cooperate with BIP to stabilize and fold proteins (20). Therefore, we wanted to determine if ALPP may be acting as a cochaperone. We aligned ALPP with other DnaJ-like cochaperone sequences and found that all DnaJ-like cochaperones, but not ALPP, contained a J-domain sequon (KYHPDK) (Fig. S7A and B). Molecular docking results suggested that no similar structural domains exist in ALPP and DnaJ-like cochaperones (Fig. S7C). Based on these findings, ALPP might not function as a DnaJ-like cochaperone.

10.1128/mBio.01716-20.7 FIG S7 Docking predictions indicate that ALPP is not a candidate DnaJ-like cochaperone. (A) Alignment of ALPP sequence with the sequences of other DNAJ-like cochaperones. (B) Multiple-sequence alignment of the J domains of 22 yeast proteins (PMID: 15170475). (C) Molecular docking and superposition results from comparisons between ALPP and DnaJ. Root mean square deviation (RMSD) value = 7.675. Download FIG S7, TIF file, 1.2 MB. Copyright © 2020 Chen et al. 2020 Chen et al. This content is distributed under the terms of the Creative Commons Attribution 4.0 International license.

### BIP chaperone activity is essential for ZIKV infection and ALPP stability.

**FIG 6:**
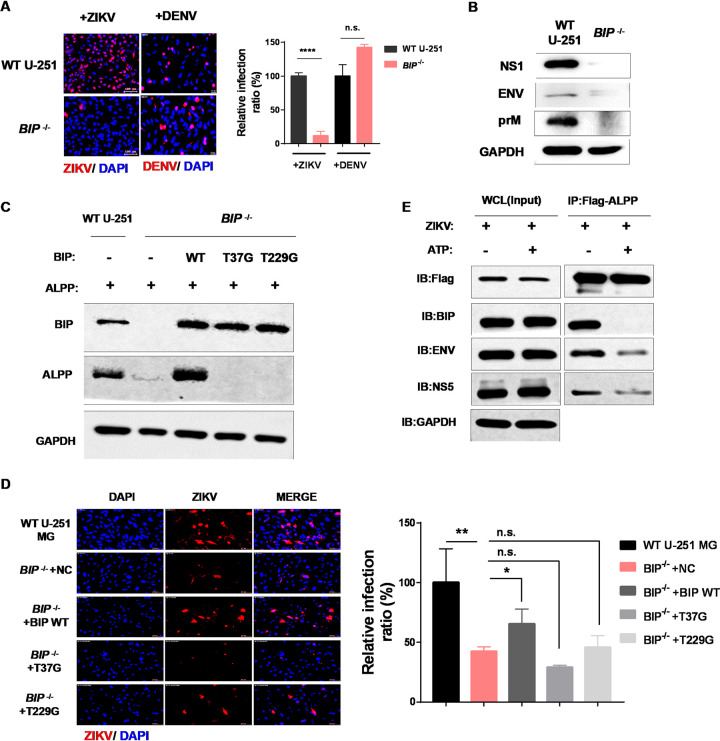
BIP chaperone activity is essential for ALPP stability and ZIKV infection. (A) Immunofluorescence (left) and statistical (right) analyses of ZIKV and DENV infection in WT and *BIP*^−/−^ U-251 MG cells. Each biological replicate (*n* = 3) contained 3,000 analyzed cells. MOI = 1. n.s., no significance; ****, *P* < 0.0001 (significantly different compared with the WT cells; two-tailed Student's *t* test). Scale bars, 10 μm. (B) Production of ZIKV proteins in WT and *ALPP*^−/−^ JEG-3 cells infected with ZIKV for 24 h. MOI = 1. (C) Western blot analysis of ALPP expression in WT and *BIP*^−/−^ U-251 MG cells. Rescue of ALPP expression by BIP reconstitution was observed with wild-type BIP but not enzymatically impaired BIP mutants (T37G and T229G). (D) Immunofluorescence (left) and statistical (right) analyses of ZIKV infection in WT and *BIP*^−/−^ U-251 MG cells overexpressing wild-type BIP or enzymatically impaired BIP mutants (T37G and T229G). MOI = 0.5. Each biological replicate (*n* = 3) contained 3,000 analyzed cells. n.s., no significance; *, *P* < 0.05; **, *P* < 0.01 (significantly different compared with the NC-treated cells; two-tailed Student's *t* test). Scale bars, 10 μm. (E) Immunoprecipitation with ALPP-2×Flag as bait followed by Western blotting with anti-Flag, anti-ZIKV ENV, anti-ZIKV NS5, or anti-BIP antibodies 24 h after ZIKV infection. MOI = 0.5. The lysates were prepared in the absence or presence of 2 mM ATP to modulate BIP substrate binding.

BIP has been implicated in the viral life cycle of yellow fever virus (YFV) and Japanese encephalitis virus (JEV) (34, 35). Here, we demonstrated that BIP depletion significantly reduced ZIKV infection but did not affect DENV infection (Fig. 6A) (20). However, the entry of ZIKV into cells was not influenced by BIP depletion (Fig. S6C), which is similar to the results found in *ALPP*^−/−^ cells. Furthermore, the expression of viral proteins, including ENV, PrM, and NS1, was significantly reduced in *BIP*^–/–^ U-251 MG cells 24 h post-ZIKV infection (Fig. 6B) and 24 h posttransfection with ZIKV proteins (Fig. S6D). These results suggest a specific role of BIP in ZIKV infection and in the stability of ZIKV proteins. We subsequently examined whether BIP also interacts with viral proteins through coimmunoprecipitation experiments using ZIKV-infected Flag-BIP-positive 293T cells (Fig. S8A) and U-251 MG cells (Fig. S8B). Similarly to the observations obtained for ALPP, we found that BIP exhibited associations with the ZIKV nonstructural proteins NS1, NS3, NS2B, and NS5 and the structural proteins ENV and C.

10.1128/mBio.01716-20.8 FIG S8 BIP directly interacts with ZIKV proteins during ZIKV infection. (A) Immunoblot and immunoprecipitation analyses of lysates of WT and BIP-overexpressing 293T cells infected with ZIKV for 24 h. (B) Immunoblot and immunoprecipitation analyses of lysates of WT and BIP-overexpressing U-251 MG cells infected with ZIKV for 24 h. Download FIG S8, TIF file, 1.6 MB. Copyright © 2020 Chen et al. 2020 Chen et al. This content is distributed under the terms of the Creative Commons Attribution 4.0 International license.

To test whether BIP chaperone activity is required for its regulation of ALPP and ZIKV infection, we reconstituted BIP-depleted cells with CRISPR-resistant wild-type BIP or ATPase-defective mutants (36). The mutants could not rescue the ALPP levels (Fig. 6C) or ZIKV infection (Fig. 6D), which suggested that BIP chaperone activity is required for ZIKV infection. Furthermore, the interaction of ALPP with ZIKV proteins or BIP was abrogated by the addition of ATP, which binds to the chaperone and induces a conformational change that releases substrates (Fig. 6E) (37).

## DISCUSSION

Clinical observations suggest that ZIKV infection in pregnant women can restrict fetal growth and cause other fetal congenital abnormalities (1, 9, 10, 13). However, to access the developing nervous system and disrupt normal neuronal development, ZIKV must penetrate and replicate in the placenta. Indeed, *in vitro* studies have shown that ZIKV can infect maternal decidual cells, first-trimester cytotrophoblasts, and extravillous trophoblasts (11, 38, 39). Previous studies have also provided evidence showing positive ZIKV replication in the placenta, the brain tissues of fetuses with microcephaly, and placenta-derived primary cells (3, 4, 6, 8–11). ALPP is primarily expressed in placental and endometrial tissues, and its expression is further upregulated during pregnancy (23–25). Here, we found that ALPP may promote high levels of ZIKV but not DENV infection by stabilizing both nonstructural and structural viral proteins together with the chaperone BIP. High levels of ZIKV activity have been detected in human and mouse placentas (3–5, 7, 8), and these are likely promoted by high levels of placental ALPP. Additionally, ALPP might also enhance ZIKV replication in other tissues, as exemplified by our findings with the U-251 MG and HK2 cell lines, which were previously confirmed to be highly permissive to ZIKV infection (28, 40). Together, our results provide evidence suggesting that ALPP exclusively facilitates ZIKV infection in placental trophoblasts and fetal brain cells, based on data obtained with JEG-3 and U-251 MG cells.

Flavivirus infection remodels the ER to form clusters of double-membrane vesicles enclosed in the vesicle packets, which contain viral replication and assembly complexes (VRACs) where viral RNA synthesis and virion assembly occur (41). The viral structural and nonstructural proteins induce the formation of VRACs (32). In addition, several host ER proteins also participate in this process (34, 42–51). Here, we demonstrated that ALPP, an ER-resident protein, interacts with and stabilizes ZIKV nonstructural proteins of the replicase complex (NS5 and NS2B-NS3 protease) and thereby orchestrates viral RNA replication. We also showed that ALPP interacted with the ZIKV envelope and capsid proteins, which suggests that ALPP is also potentially involved in virion assembly (21, 32).

ALPP is a homodimeric enzyme, and each catalytic site contains three metal ions (two Zn ions and one Mg ion) that are necessary for enzymatic activity. Mutations of the enzymatic activity sites affect the hydrophobic pocket formation of ALPP and its function (33). Intriguingly, we found that the phosphatase activity of ALPP was likely required for optimal ZIKV activity. Previous studies uncovered that differentially phosphorylated states of flavivirus NS5 affected its subcellular localization and interaction with NS3, controlling its function in the viral RNA replicase (52, 53). Moreover, dephosphorylation of West Nile virus capsid protein enhances the processes of nucleocapsid assembly (54). Thus, we hypothesize that the phosphatase activity of ALPP may control the phosphorylation status of ZIKV proteins and may regulate the aggregation, interaction, stability, and function of ZIKV proteins in viral RNA replicase. Dynamic phosphorylation of ZIKV structural proteins may also modulate viral assembly and maturation. Finally, the enzymatic activity of ALPP could also facilitate its proper structural conformation and interaction with BIP, which stabilizes ALPP during ZIKV infection. In the future, more studies are needed to further understand how ALPP and its enzymatic activity precisely regulate ZIKV life cycle.

The proper folding and assembly of viral proteins might be mediated by host chaperones. Previous studies have shown that cytosolic Hsp70 isoforms (including HSPA1A, HSPA1B, and HSPA8) are needed at distinct steps of the flavivirus viral cycle, including entry, RNA replication, and biogenesis of virions (20, 43). During JEV infection (55), BIP is needed for entry and formation of the replication complex. In this study, we demonstrated that BIP cooperates with ALPP and plays roles exclusively in ZIKV infection and not in DENV infection. Given the high degree of similarity between flavivirus members, the requirement of distinct isoforms of Hsp70 in various viral functions is intriguing. In this study, we identified a specific BIP-ALPP partnership due to the strong and specific binding of these proteins in placental cells. Our data further demonstrate that the specific requirement of BIP by ZIKV is likely endowed by its partner, ALPP. In fact, in the absence of ALPP, BIP itself cannot ensure the stability of ZIKV proteins and fails to support ZIKV infection. Thus, our findings provide a new model regarding how chaperone proteins can function specifically during flavivirus infections. Interestingly, antibodies directed against both the N and C termini of BIP suppress both the binding of the DENV to the cell surface and the infectivity of this virus in HepG2 cells (56). Our data raise the possibility that ALPP or BIP can be targeted to intervene in ZIKV infection.

Taken together, the results indicate that identification of the host factors that play important roles in regulating the ZIKV life cycle provides novel therapeutic targets for the development of antiflavivirus drugs. ALPP might be targeted to block ZIKV infection in the placenta and to ameliorate microcephaly. These possibilities should be explored further in a mouse model. An in-depth understanding of virus mechanisms and biology will facilitate the development of antiviral antibodies, vaccine therapies, and virus-targeted small molecules.

## MATERIALS AND METHODS

### Cell lines and viruses.

Aedes albopictus C6/36 cells were grown in a mixture of 30% RPMI 1640 (Gibco) and 60% Dulbecco’s modified Eagle’s medium (DMEM; Gibco) supplemented with 10% fetal bovine serum (FBS; Gibco). The U-251 MG cell line was purchased from BeNa Culture Collection (BNCC), and the 293T, HK2, and JEG-3 cell lines were purchased from American Type Culture Collection (ATCC). The 293T and U-251 MG cells were cultured in DMEM (Gibco) supplemented with 10% FBS, 100 IU/ml penicillin, and 100 μg/ml streptomycin. The BIP-KO cells were cultured in DMEM (Gibco) supplemented with 15% FBS, and the JEG-3 cells were cultured in minimal essential medium (MEM) (Gibco) supplemented with 10% FBS, 100 IU/ml penicillin, and 100 μg/ml streptomycin. The HK2 cells were cultured in DMEM/F12 (Gibco) (1:1) supplemented with 10% FBS, 100 IU/ml penicillin, and 100 μg/ml streptomycin. The 293T, U-251 MG, JEG-3, and HK2 cells were maintained at 37°C, and the C6/36 cells were maintained at 28°C in a fully humidified atmosphere containing 5% CO_2_. All the cell lines were tested by Saily Bio (Shanghai, China) and were free of mycoplasma contamination. The ZIKV MR766 stock was purchased from ATCC (ATCC VR-1838). The ZIKV SZ01 virus strain (GenBank accession number KU866423) was kindly provided by Cheng-Feng Qin at the Department of Virology, State Key Laboratory of Pathogen and Biosecurity, Beijing Institute of Microbiology and Epidemiology.

### Plasmids and molecular cloning.

pCMV-Zika-ENV, NS1, NS2B, NS3, NS4A, NS4B, and NS5 natural native open reading frame (ORF) mammalian expression plasmids were purchased from Sino Biological. pcDNA3.1+/C-(K)DYK-ALPP and pcDNA3.1+/C-(K)DYK-BIP mammalian expression plasmids were purchased from GenScript. To generate the C-terminal 2×Flag-tagged ALPP lentiviral construct (pHAGE-ALPP-2×Flag), pHAGE-CMV-2×Flag-IRES-puro was digested with NotI and XhoI. Full-length ALPP was then amplified from pcDNA3.1(+)-ALPP-DYK. These two fragments were homologously recombined using a ClonExpress II One Step cloning kit (Vazyme Biotech, C112-02) according to the manufacturer’s instructions to generate the final pHAGE-ALPP-2×Flag construct. Similarly, the BIP-tagged lentiviral construct was generated by replacing ALPP with the complete BIP ORF to generate pHAGE-BIP-2×Flag.

### CRISPR-Cas9 cloning.

Oligonucleotides encoding sgRNAs for generating KO cells using CRISPR-Cas9 were cloned into lentiCRISPRv2 plasmid (Addgene Plasmid, 52961) as previously described (28). The oligonucleotide sequences of the sgRNAs targeting ALPP and BIP are listed in Table S1B in the supplemental material. LentiCRISPRv2 clones containing the guide sequences were sequenced and purified and used for lentiviral production. To generate heterogeneous KO cell populations, JEG-3, U-251 MG, or HK2 cells were infected with the lentiCRISPRv2-derived lentivirus for 36 h and then reseeded into complete DMEM containing 1 to 4 μg/ml puromycin for 14 days to select for the transduced cells. Single-cell clones of JEG-3 cells targeted for CRISPR-mediated ALPP KO were diluted with the parental JEG-3 cells at a ratio of 1/1,000. One of the cells from the cell mixtures was plated in each well of a 96-well plate. Once the cells reached confluence, the cells were passaged based on a 48-well format in the presence of 4 μg/ml puromycin to kill nontargeted cells. The surviving populations derived using this approach were propagated and expanded for 4 weeks prior to cryopreservation of the stock cultures. Using this strategy, an ALPP-targeted KO cell line (*ALPP*^−/−^) and a BIP-targeted KO cell line (*BIP*^−/−^) were generated, and the gene KO efficiency was analyzed through immunoblot or immunofluorescence assays.

### ZIKV infection.

Prior to ZIKV infection, U-251 MG, JEG-3, and HK2 cells were seeded in 24-well plates (2 × 10^4^ cells per well) or 6-well plates (1 × 10^5^ cells per well). Twenty-four hours after seeding, the cells were rinsed once with phosphate-buffered saline (PBS) and then incubated with ZIKV at the indicated multiplicity of infection (MOI) in serum-free medium for 1 h at 37°C unless otherwise noted. The ZIKV-containing medium was then replaced with fresh DMEM or MEM supplemented with 2% FBS.

For the immunofluorescence assay, cells were seeded in four- or eight-well glass Millicell EZ Slide chambers (Millipore) at 4 × 10^4^ or 2 × 10^4^ cells per well and then infected with ZIKV as described above. After incubation at 37°C for the indicated times, the cells were rinsed twice with phosphate-buffered saline (PBS) and fixed with 4% paraformaldehyde (PFA).

For the plaque assay, 1 × 10^5^ Vero cells were seeded in 12-well plates. The next day, monolayer cells were infected for 2 h with serial dilutions of the supernatant collected from WT and ALPP-KO U-251 cells at 24, 48, and 72 h. The inoculum was then removed, and the cells were incubated in medium containing 2% FBS and 0.5% agarose. The cell monolayer was fixed with 10% formalin and stained with 1% crystal violet, and the plaques were counted after 5 days.

### Immunoprecipitation assay.

We used two approaches for the coimmunoprecipitation experiments. In the first approach, WT and ALPP- or BIP-expressing cells were mock infected or infected with ZIKV for 24 h. In the second approach, U-251-2×Flag control cells or U-251-ALPP-2×Flag cells (2 × 10^6^ cells per 10-cm-diameter dish) were transiently transfected with 14 μg of pCMV-NS5 or pCMV-E separately using TurboFect transfection reagent (Thermo Fisher Scientific). The cells were rinsed twice with cold PBS, transferred to clean tubes, and lysed in cell buffer supplemented with 1% protease inhibitor cocktail (Sigma, catalog number P8340) for Western blotting and immunoprecipitation. The cell lysates were incubated with Pierce protein A/G agarose (Sigma, 20422) for 4 h at 4°C and then centrifuged at 10,000 × *g* and 4°C for 10 min. When used, ATP was included at a final concentration of 2 mM (Selleck, S1985). The supernatant was transferred to a new tube and incubated with 30 μl of anti-Flag M2 affinity gel (Sigma, A2220) overnight at 4°C. The Sepharose samples were centrifuged and then washed five times with cell lysis buffer and eluted using 3×Flag peptide (Sigma, F4799). All the samples were then boiled with SDS loading buffer for 10 min.

### Immunofluorescence and confocal microscopy.

Cells on slides were fixed with 4% paraformaldehyde (PFA) for 20 min at room temperature, permeabilized with 0.1% Triton X-100–PBS for 5 min, and blocked with blocking buffer (1% BSA–2% donkey serum–PBS) for 30 min. Immunofluorescence analyses of ZIKV-infected cells were performed using a mouse anti-flavivirus envelope protein antibody (Millipore, clone D1-4G2-4-15) (1:300), rabbit anti-flavivirus capsid protein antibody (GeneTex, GTX133317) (1:1,000), rabbit anti-flavivirus NS2B protein antibody (GeneTex, GTX133308) (1:1,000), rabbit anti-flavivirus envelope protein antibody (GeneTex, GTX133314) (1:1,000), and either Alexa Fluor 568-conjugated donkey anti-mouse IgG (H+L) (Abcam, ab175472) (1:1,000) or Alexa Fluor 488-conjugated donkey anti-rabbit IgG (H+L) (Abcam, ab150077) (1:1,000).

For ALPP and BIP detection, ALPP-U-251 MG cells were incubated first with mouse anti-ALPP antibody (R&D, MAB59051) (1:100) or rabbit anti-BIP antibody (ABclonal, A0241) (1:200) as appropriate and then with an Alexa Fluor-conjugated secondary antibody (AF-488 for visualization in the green channel and AF-568 for visualization in the red channel). All the cells were mounted with ProLong Gold Antifade with DAPI (4′,6-diamidino-2-phenylindole) (Life Technologies, P36931) and imaged using a TissueFAXS 200 flow-type tissue cytometer (TissueGnostics GmbH, Vienna, Austria). Some images were acquired with a Zeiss Axio Imager Z2 microscope (Wetzlar, Germany) using a 63×oil immersion lens objective and a Hamamatsu ORCA-Flash4.0 scientific complementary metal oxide semiconductor (sCMOS) camera (Hamamatsu City, Japan). All the statistical analyses of the immunofluorescence staining data were performed using the results from at least 3,000 cells per replicate, and the data are shown as means ± standard errors of the means (SEMs).

### Viral entry assay.

ZIKV cell entry experiments were performed based on a previously described protocol (28). In brief, for the virus binding assay, 1 × 10^5^ cells were seeded in a 12-well plate, cultured for 24 h, and infected with ZIKV (MOI = 20) in cold MEM on ice for 1 h. Any unbound virus was removed, and the cells were then washed twice with 1× PBS. The cells were incubated with prewarmed medium for 1 h at 37°C to initiate ZIKV internalization, and cell lysates were then harvested for vRNA quantitation by quantitative real-time PCR (RT-qPCR).

### RT-qPCR.

RNA was extracted using an RNeasy RNA isolation kit (Qiagen) according to the manufacturer’s instructions. One microgram of RNA was transcribed into cDNA using random primers and Moloney murine leukemia virus reverse transcriptase (Promega, Charbonnieres, France). RT-qPCR was performed using the resulting cDNA templates and GoTaq qPCR master mix (Promega) with an Applied Biosystems 7300 real-time PCR cycler (see the supplemental material for the primer sequences). The PCR data were analyzed using SDS software (Applied Biosystems), and GAPDH (glyceraldehyde-3-phosphate dehydrogenase) expression was used as an internal control. For the ZIKV infection experiments, the expression of the genes of interest in the infected cells was normalized to that in unstimulated WT cells unless otherwise noted. All presented RT-qPCR data represent results from four biological replicates.

### Western blot (WB) analysis.

The cells were lysed with 4× SDS loading buffer and denatured at 95°C for 10 min. The protein samples were resolved by SDS-PAGE, transferred to polyvinylidene difluoride (PVDF) membranes (GE Healthcare), and processed for Western blot (WB) analysis. WB detection of ALPP and BIP was performed using rabbit anti-ALPP antibody (ABclonal, A6353) (1:1,000) and anti-BIP antibody (GeneTex, GTX102580) (1:1,000) as the primary antibodies and goat anti-rabbit IgG-horseradish peroxidase (IgG-HRP) antibody (Santa Cruz Biotechnology, B2615) (1:3,000) as the secondary antibody, and β-actin or GAPDH was used as a loading control.

Rabbit-derived antiviral protein antibodies, including anti-NS1 (GeneTex, GTX133307) (1:2,000), anti-NS2b (GeneTex, GTX133308) (1:2,000), anti-NS5 (GeneTex, GTX133312) (1:2,000), anti-NS3 (GeneTex, GTX133309) (1:2,000), anti-envelope (GeneTex, GTX133314) (1:2,000), and anti-PrM (GeneTex, GTX133305) (1:2,000) antibodies, were used as primary antibodies for detecting ZIKV proteins.

The other antibodies used in the study included anti-Flag M2 (Sigma, F1804) (1:2,000), anti-beta actin (ABclonal, AC026) (1:1,000), anti-GAPDH (ABclonal, A19056) (1:1,000), goat anti-rabbit IgG-HRP (Santa Cruz Biotechnology, B2615) (1:3,000), and goat anti-rabbit IgG-HRP (Invitrogen, 31430) (1:5,000) antibodies.

### Viral titration.

U-251 MG cells were seeded in 96-well plates at 3 × 10^3^ cells per well. Twenty-four hours later, the supernatant, harvested at the indicated time points postinfection, was serially diluted 10-fold (from 10^−1^ to 10^−6^) with fresh DMEM supplemented with 2% FBS and added to U-251 MG cells, and the cells were then incubated for 1 h at 37°C. Eight replicates of each dilution were prepared. The ZIKV-containing medium was then replaced with fresh DMEM supplemented with 2% FBS. The cell morphology was observed daily. The data were calculated and analyzed using the Reed-Muench method or the Spearman-Karber method.

### Statistical analyses.

For quantification of the infected cells, at least 1,000 fluorescent cells were imaged and counted using a flow-type tissue cytometer. Three replicates were established per sample. The data are presented as means ± SEMs. Significant differences between groups were determined using a two-tailed Student's *t* test unless otherwise noted. For the RT-qPCR analyses, at least four biological replicates were established for each sample. RT-qPCR-derived values that were 10-fold higher or lower than the mean values were identified as outliers and excluded from the analyses. The data are presented as means ± SEMs, and significant differences between groups were determined by two-way analysis of variance (ANOVA) with the Sidak multiple-comparison test unless otherwise noted.
