# Supplementary figures and images for: Placental Alkaline Phosphatase Promotes Zika Virus Replication by Stabilizing Viral Proteins through BIP
Source: mBio. 2020 Sep 15;11(5):e01716-20. doi: 10.1128/mBio.01716-20 (PMC7492734; doi:10.1128/mBio.01716-20)

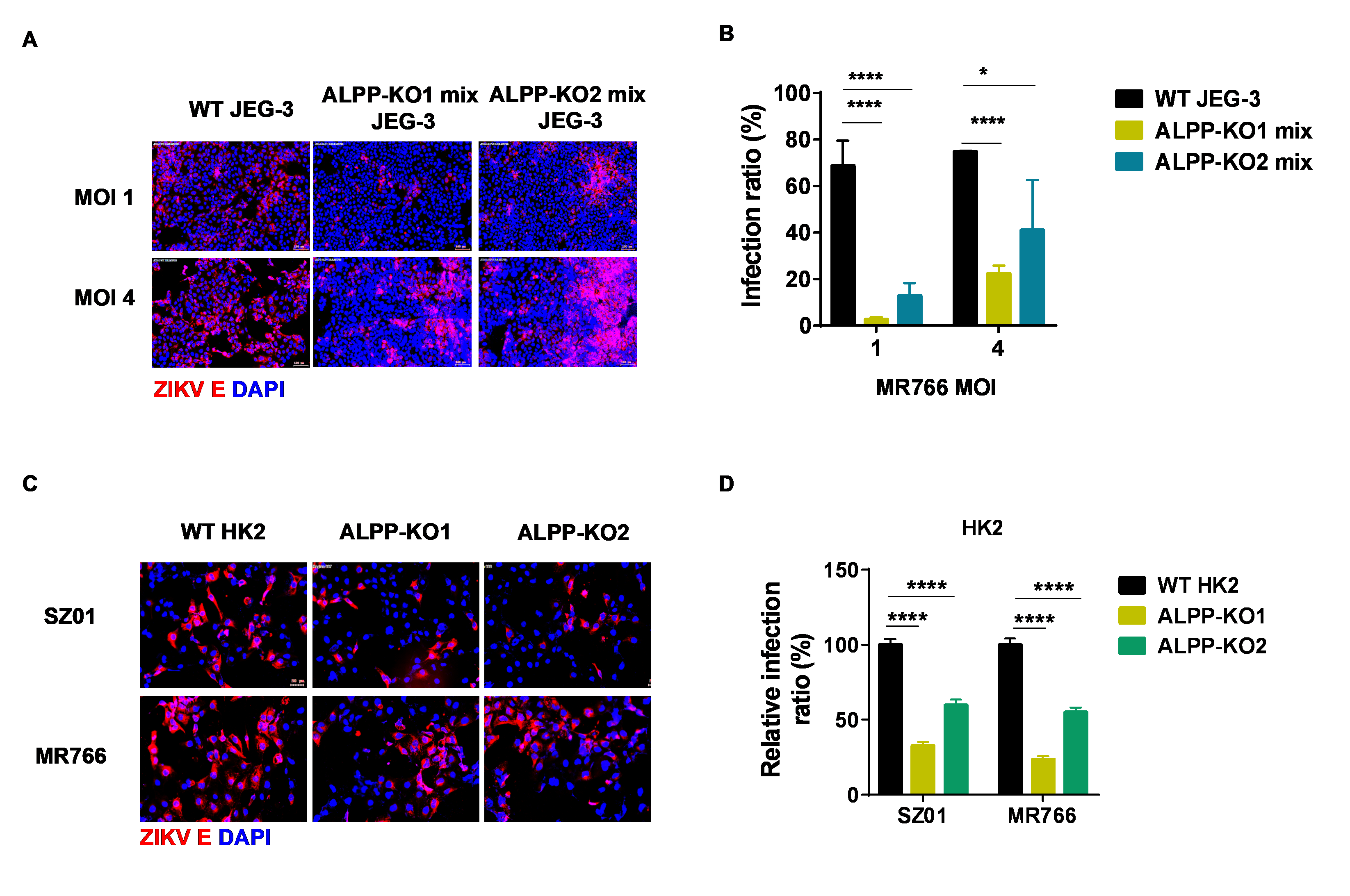

Supplement: FIG S2 [file mBio.01716-20-sf002.tif]

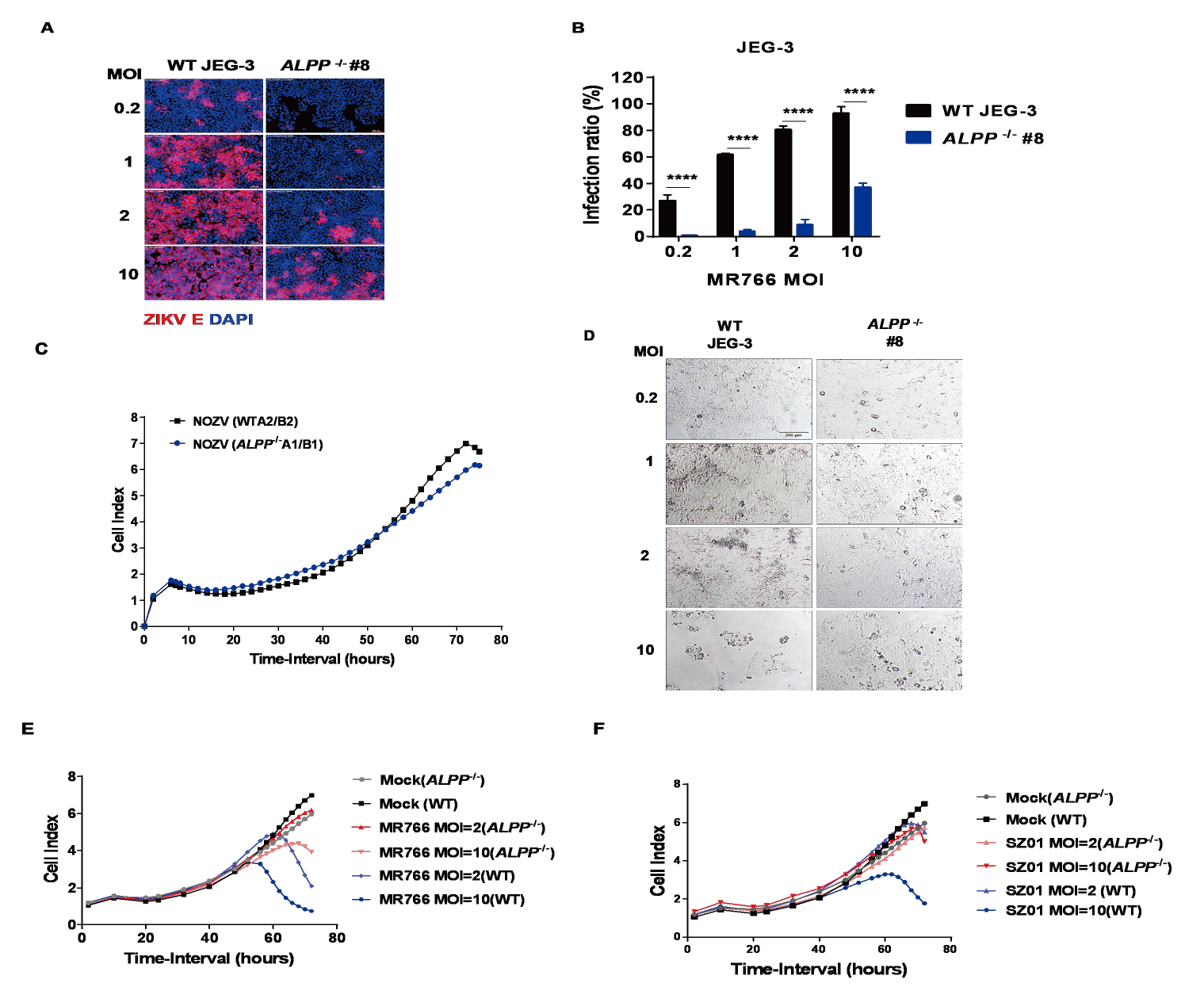

Supplement: FIG S3 [file mBio.01716-20-sf003.tif]

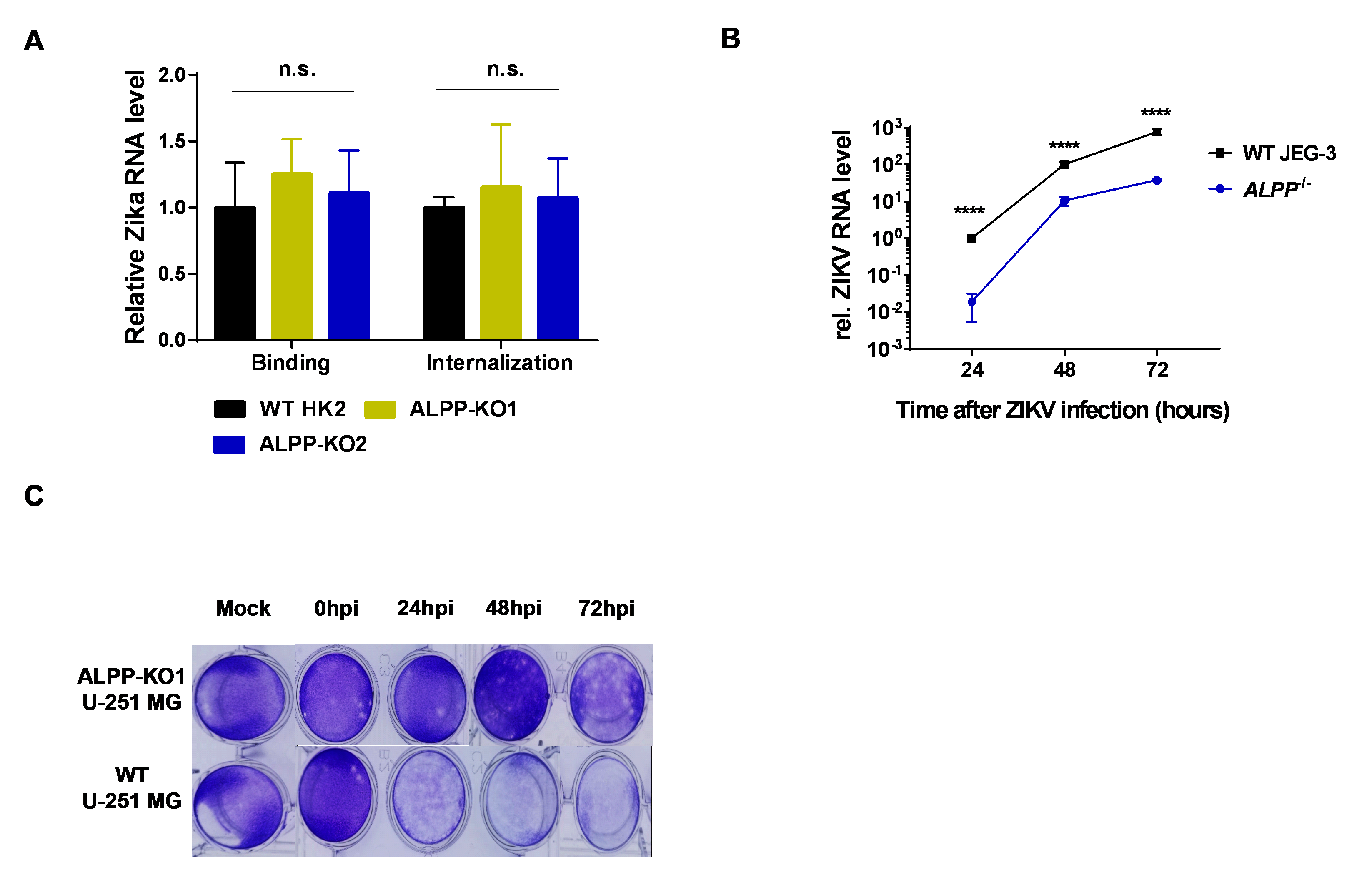

Supplement: FIG S4 [file mBio.01716-20-sf004.tif]

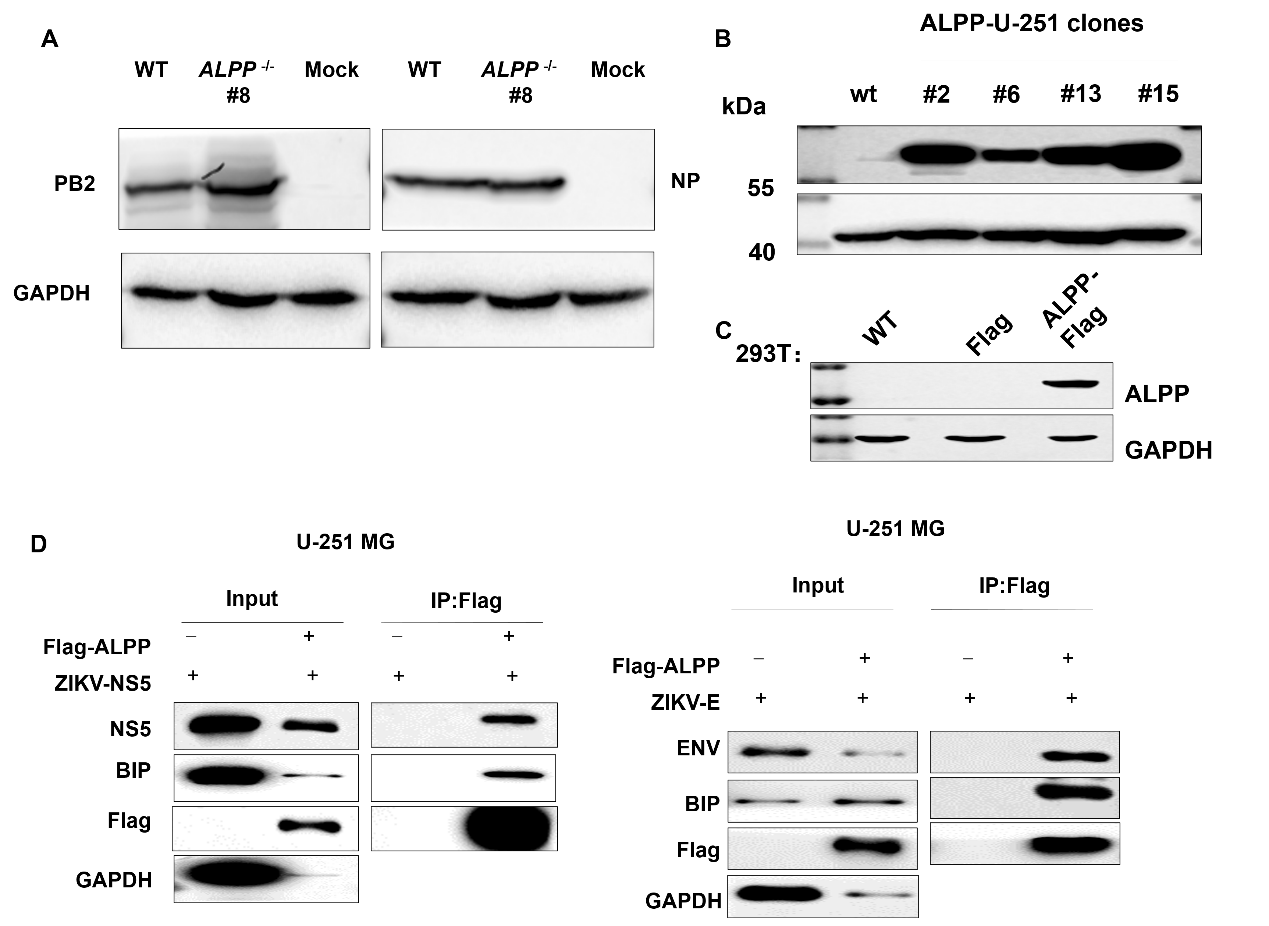

Supplement: FIG S5 [file mBio.01716-20-sf005.tif]

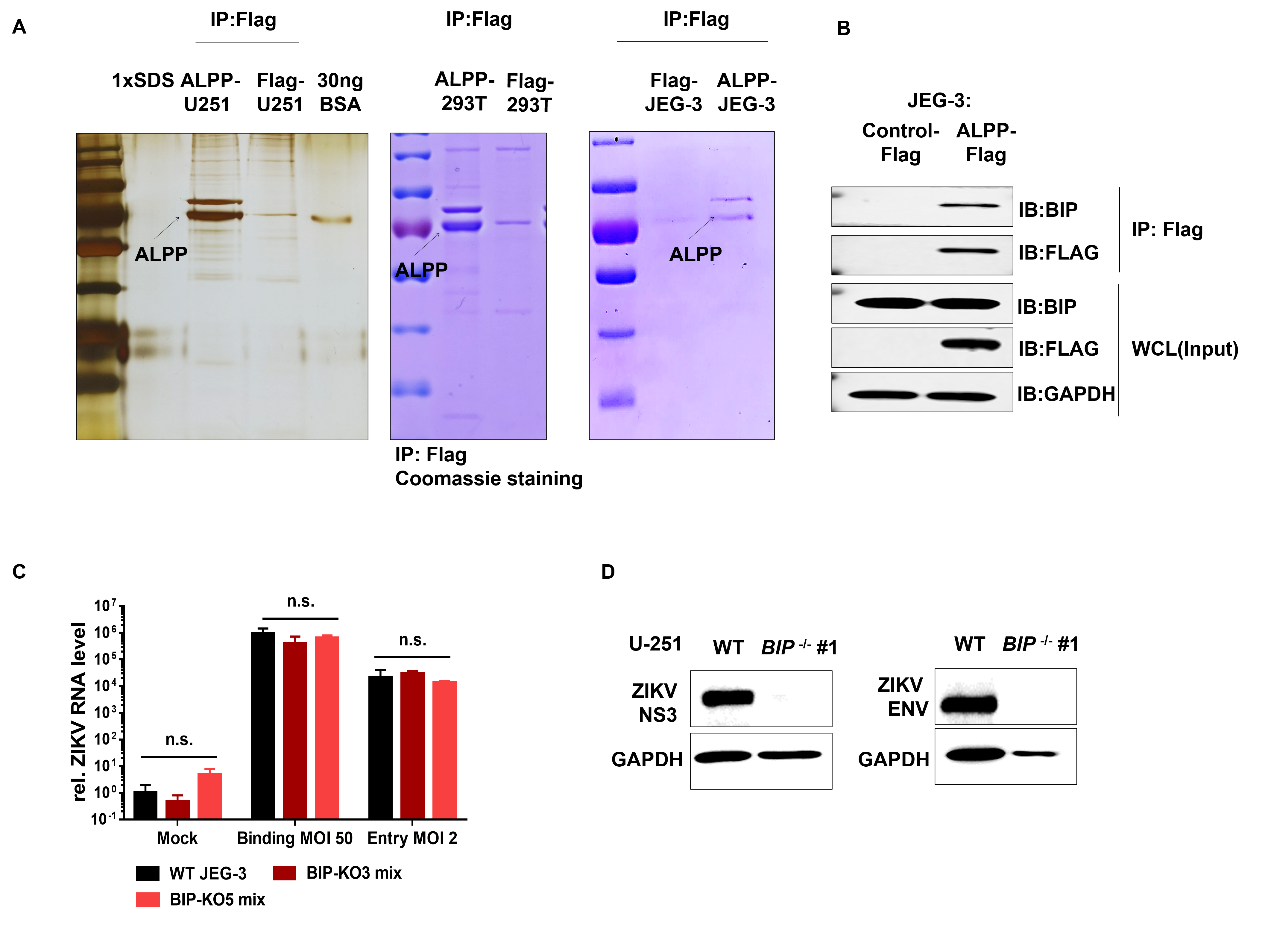

Supplement: FIG S6 [file mBio.01716-20-sf006.tif]

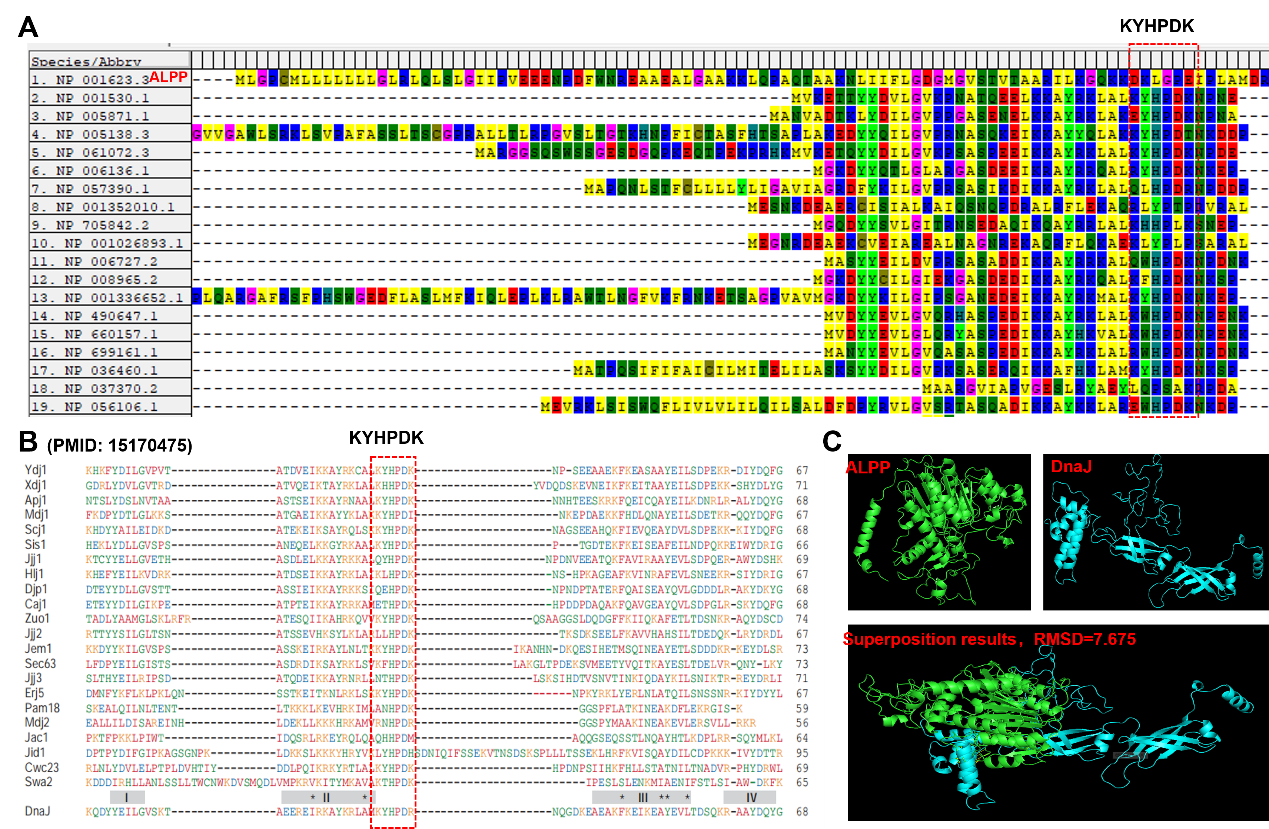

Supplement: FIG S7 [file mBio.01716-20-sf007.tif]

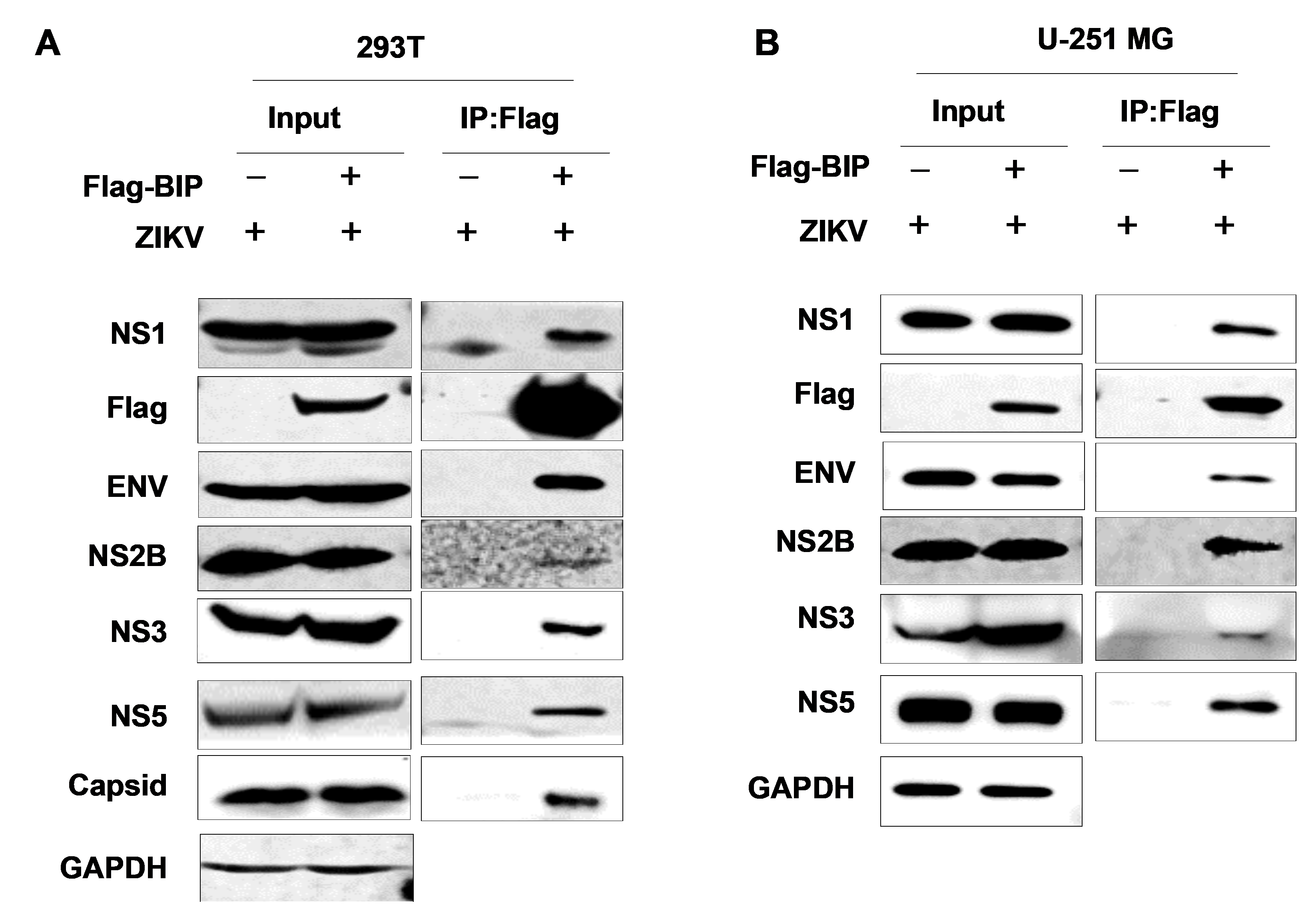

Supplement: FIG S8 [file mBio.01716-20-sf008.tif]
