# Supplementary material for: Placental Alkaline Phosphatase Promotes Zika Virus Replication by Stabilizing Viral Proteins through BIP
Source: mBio. 2020 Sep 15;11(5):e01716-20. doi: 10.1128/mBio.01716-20 (PMC7492734; doi:10.1128/mBio.01716-20)
Supplement: TABLE S1 [file mBio.01716-20-st001.docx]

**Supplementary Table1**

**Table S1A.** List of primers.

| Experiments | Primer name | Primer sequence |
| --- | --- | --- |
| sgRNA synthesis | ALPP-F1 | CACCGCATGGACCGCTTCCCATATG |
| sgRNA synthesis | ALPP-R1 | AAACCATATGGGAAGCGGTCCATGC |
| sgRNA synthesis | ALPP-F2 | CACCGCACGAGTGCAGCACGCCTCG |
| sgRNA synthesis | ALPP-R2 | AAACCGAGGCGTGCTGCACTCGTGC |
| sgRNA synthesis | BIP-F1 | CACCGCGACATAGGACGGCGTGATG |
| sgRNA synthesis | BIP-R1 | AAACCATCACGCCGTCCTATGTCGC |
| sgRNA synthesis | BIP-F3 | CACCGCGGTGGTCGGCATCGACCTG |
| sgRNA synthesis | BIP-R3 | AAACCAGGTCGATGCCGACCACCGC |
| sgRNA synthesis | BIP-F5 | CACCGAATGGCAAGGAACCATCCCG |
| sgRNA synthesis | BIP-R5 | AAACCGGGATGGTTCCTTGCCATTC |
| RT-qPCR | ALPP-F | ACTGGGGCCTGAGATACCC |
| RT-qPCR | ALPP-R | TCGTGTTGCACTGGTTAAAGC |
| RT-qPCR | ZIKV-F1 | TGGGAGGTTTGAAGAGGCTG |
| RT-qPCR | ZIKV-R1 | TCTCAACATGGCAGCAAGATCT |
| RT-qPCR | ZIKV-F2 | CATATTCCTTGTGCACCGCG |
| RT-qPCR | ZIKV-R2 | GCATACTGCACCTCCACTGT |
| RT-qPCR | GAPDH-F | CTGGGCTACACTGAGCACC |
| RT-qPCR | GAPDH-R | AAGTGGTCGTTGAGGGCAATG |
| Plasmid construction | ALPP2Flag-pHAGE-HrF | CTCCATAGAAGACACCGGCGGCCGCCACCATGCTGGGGCCCTGCATGCT |
| Plasmid construction | ALPP2Flag-pHAGE-HrR | CATCATCATCCTTATAATCCTCGAGGGGAGCAGTGGCCGTCT |
| Plasmid construction | BIP2Flag-pHAGE-HrF | ACCTCCATAGAAGACACCGATGAAGCTCTCCCTGGTGGC |
| Plasmid construction | BIP2Flag-pHAGE-HrR | CATCATCATCCTTATAATCCAACTCATCTTTTTCTGCTG |

**Table S1B.** Proteins identified by liquid chromatography-mass spectrometry (LC-MS).

| Master Protein Accession | Description | Coverage [%] | # Peptides | # PSMs | # Unique Peptides | # AAs | MW [kDa] | calc. pI | Score Sequest HT: Sequest HT | # Protein Groups |
| --- | --- | --- | --- | --- | --- | --- | --- | --- | --- | --- |
| P11021 | Endoplasmic reticulum chaperone BIP OS=Homo sapiens OX=9606 GN=HSPA5 PE=1 SV=2 | 43 | 26 | 468 | 24 | 654 | 72.3 | 5.16 | 1286.17 | 1 |
| P05187 | Alkaline phosphatase, placental type OS=Homo sapiens OX=9606 GN=ALPP PE=1 SV=2 | 33 | 14 | 44 | 14 | 535 | 57.9 | 6.29 | 115.37 | 1 |
| P14923 | Junction plakoglobin OS=Homo sapiens OX=9606 GN=JUP PE=1 SV=3 | 19 | 12 | 25 | 11 | 745 | 81.7 | 6.14 | 66.89 | 1 |
| P15924 | Desmoplakin OS=Homo sapiens OX=9606 GN=DSP PE=1 SV=3 | 7 | 21 | 29 | 21 | 2871 | 331.6 | 6.81 | 67.03 | 1 |
| P60709 | Actin, cytoplasmic 1 OS=Homo sapiens OX=9606 GN=ACTB PE=1 SV=1 | 36 | 9 | 62 | 9 | 375 | 41.7 | 5.48 | 142.74 | 1 |
| E9PKE3 | Heat shock cognate 71 kDa protein OS=Homo sapiens OX=9606 GN=HSPA8 PE=1 SV=1 | 16 | 8 | 22 | 1 | 627 | 68.8 | 5.52 | 54.13 | 1 |
| P13667 | Protein disulfide-isomerase A4 OS=Homo sapiens OX=9606 GN=PDIA4 PE=1 SV=2 | 11 | 6 | 9 | 6 | 645 | 72.9 | 5.07 | 28.38 | 1 |
| Q13885 | Tubulin beta-2A chain OS=Homo sapiens OX=9606 GN=TUBB2A PE=1 SV=1 | 22 | 8 | 16 | 4 | 445 | 49.9 | 4.89 | 40.58 | 1 |
| P54652 | Heat shock-related 70 kDa protein 2 OS=Homo sapiens OX=9606 GN=HSPA2 PE=1 SV=1 | 12 | 6 | 19 | 2 | 639 | 70 | 5.74 | 42.43 | 1 |
| P33897 | ATP-binding cassette subfamily D member 1 OS=Homo sapiens OX=9606 GN=ABCD1 PE=1 SV=2 | 11 | 6 | 10 | 6 | 745 | 82.9 | 8.95 | 27.16 | 1 |
